# Introduction of High Throughput Magnetic Resonance T2-Weighted Image Texture Analysis for WHO Grade 2 and 3 Gliomas

**DOI:** 10.1371/journal.pone.0164268

**Published:** 2016-10-07

**Authors:** Manabu Kinoshita, Mio Sakai, Hideyuki Arita, Tomoko Shofuda, Yasuyoshi Chiba, Naoki Kagawa, Yoshiyuki Watanabe, Naoya Hashimoto, Yasunori Fujimoto, Toshiki Yoshimine, Katsuyuki Nakanishi, Yonehiro Kanemura

**Affiliations:** 1 Department of Neurosurgery, Osaka Medical Center for Cancer and Cardiovascular Diseases, Osaka, Japan; 2 Department of Radiology, Osaka Medical Center for Cancer and Cardiovascular Diseases, Osaka, Japan; 3 Department of Neurosurgery, Osaka University Graduate School of Medicine, Suita, Japan; 4 Division of Stem Cell Research, Institute for Clinical Research, Osaka National Hospital, Osaka, Japan; 5 Department of Radiology, Osaka University Graduate School of Medicine, Suita, Japan; 6 Department of Neurosurgery, Kyoto Prefectural University Graduate School of Medical Science, Kyoto, Japan; 7 Division of Regenerative Medicine, Institute for Clinical Research, Osaka National Hospital, Osaka, Japan; 8 Department of Neurosurgery, Osaka National Hospital, Osaka, Japan; University of Pittsburgh School of Medicine, UNITED STATES

## Abstract

Reports have suggested that tumor textures presented on T2-weighted images correlate with the genetic status of glioma. Therefore, development of an image analyzing framework that is capable of objective and high throughput image texture analysis for large scale image data collection is needed. The current study aimed to address the development of such a framework by introducing two novel parameters for image textures on T2-weighted images, i.e., Shannon entropy and Prewitt filtering. Twenty-two WHO grade 2 and 28 grade 3 glioma patients were collected whose pre-surgical MRI and *IDH1* mutation status were available. Heterogeneous lesions showed statistically higher Shannon entropy than homogenous lesions (*p* = 0.006) and ROC curve analysis proved that Shannon entropy on T2WI was a reliable indicator for discrimination of homogenous and heterogeneous lesions (*p* = 0.015, AUC = 0.73). Lesions with well-defined borders exhibited statistically higher Edge mean and Edge median values using Prewitt filtering than those with vague lesion borders (*p* = 0.0003 and *p* = 0.0005 respectively). ROC curve analysis also proved that both Edge mean and median values were promising indicators for discrimination of lesions with vague and well defined borders and both Edge mean and median values performed in a comparable manner (*p* = 0.0002, AUC = 0.81 and *p* < 0.0001, AUC = 0.83, respectively). Finally, *IDH1* wild type gliomas showed statistically lower Shannon entropy on T2WI than *IDH1* mutated gliomas (*p* = 0.007) but no difference was observed between *IDH1* wild type and mutated gliomas in Edge median values using Prewitt filtering. The current study introduced two image metrics that reflect lesion texture described on T2WI. These two metrics were validated by readings of a neuro-radiologist who was blinded to the results. This observation will facilitate further use of this technique in future large scale image analysis of glioma.

## Introduction

Recent large scale collection of glioma data has made it possible to identify prognostic or predictive genetic alterations of the tumor. These alterations include changes in the *MGMT* promoter methylation status for glioblastoma [1], *IDH1/2* mutation, *TERT* promoter mutation and 1p/19q co-deletion for WHO grade 2 and 3 gliomas [2,3], and they have had a major impact on both standard treatment of care and on the design of randomized clinical trials for this malignant disease [4]. These findings have also driven the neuro-oncology community to explore the possibility of “molecular imaging” of genetic alterations of glioma using various modalities, with magnetic resonance imaging (MRI) being the major role player [5–7]. Characteristic features of the tumor such as different tumor locations within the brain according to the tumor genetic alteration status have already been reported using a voxel-based tumor location analyzing technique; Analysis of Differential Involvement (ADIFFI) [8–10], or by identifying the main anatomical structure(s) involved by the tumor using a large sample size [11]. For example, it has been clearly shown that *IDH* mutated glioblastoma arises at the frontal lobe [8] or that *H3F3A* K27M mutated glioma arises at the thalamus [12]. Although such location analysis can readily be performed in a rather objective manner, the texture of the images of each tumor cannot be easily analyzed, which eliminates subjective assessment of the observer. There have been reports suggesting that tumor textures presented on T2-weighted images (T2WI), such as tumor heterogeneity and tumor border diffuseness, correlate with the genetic status of glioma [13]. It is therefore desirable to develop an image analyzing framework that is capable of objective and high throughput image texture analysis for large scale image data collection of glioma, similar to voxel-based tumor location analysis such as ADIFFI, in order to confirm and validate previous finding based on a rather small sample size and subjective analysis of images. The current study aimed to address the development of such a framework by introducing two novel parameters that have the potential to objectively analyze image textures on T2WI, i.e., Shannon entropy and Prewitt filtering, which could reflect image heterogeneity and lesion border sharpness, respectively, for WHO grade 2 and 3 gliomas. Image analysis workflow will be described and the obtained numeric data was validated by subjective assessment of the images that were read by a neuro-radiologist blinded to the numeric data.

## Materials and Methods

### Patient selection

This study was approved by the local ethics committee (Ethics committees at Osaka Medical Center for Cancer and Cardiovascular Diseases, Osaka University Graduate School of Medicine and Osaka National Hospital) and was found to conform to generally accepted scientific principles and ethical standards. Participants provided written informed consent to participate in this study and medical record data and images were collected for this study in a fully anonymized and de-identified form. We recruited 22 WHO grade 2, and 28 grade 3 glioma patients who were diagnosed by local board-certified pathologists according to the WHO classification and whose pre-surgical MRI and *DH1* mutation status were available. Genetic analyses were performed with written informed consent. Detailed patient characteristics are presented in the Table 1.

**Table 1:** Patient characteristics.

Case number	Age	Sex	Diagnosis	Location	WHO grade	*IDH* status		Lesion heterogeniety	T2WI entropy	Lesion edge diffuseness	Edge (mean)	Edge (median)
1	22	F	DA	Lt. Frontal—Insular	2	mt	***	homo	6.17	well defined	33.47	28.64
2	24	M	DA	Lt. Temporal	2	wt	*	hetero	6.13	vague	27.54	24.08
3	25	M	DA	Lt. Temporal	2	wt	***	hetero	5.73	well defined	23.64	21.38
4	26	F	DA	Lt. Putamen	2	wt	**	homo	5.74	well defined	50.28	50.96
5	30	F	DA	Lt. Temporal	2	mt	***	hetero	6.10	vague	31.89	21.63
6	31	M	DA	Rt. Temporal—Insular	2	mt	***	hetero	6.25	well defined	52.37	42.98
7	40	M	DA	Lt. Frontal	2	mt	***	homo	5.69	well defined	34.48	29.43
8	42	F	DA	Lt. Frontal	2	wt	***	homo	5.59	well defined	25.62	21.56
9	42	F	DA	Lt. Insular—Temporal	2	mt	***	hetero	5.82	vague	28.37	22.00
10	46	M	DA	Rt. Frontal	2	mt	**	homo	5.79	well defined	38.06	35.36
11	49	F	DA	Rt. Thalamus	2	wt	**	homo	5.77	vague	19.35	16.13
12	64	M	DA	Rt. Temporal—Occipital	2	mt	***	hetero	7.06	vague	33.09	24.20
13	65	F	DA	Rt. Frontal—Insular—Temporal	2	mt	***	hetero	5.76	vague	15.98	12.21
14	67	M	DA	Lt. Temporal—Insular	2	mt	***	hetero	5.43	well defined	31.40	24.84
15	44	M	GG	Rt. Temporal	2	wt	**	homo	6.40	well defined	82.86	73.35
16	33	M	OA	Lt.Frontal	2	mt	**	homo	6.34	vague	32.94	28.93
17	36	M	OA	Rt. Frontal	2	mt	***	hetero	5.86	vague	23.93	19.00
18	36	F	OL	Lt. Frontal	2	mt	**	hetero	5.85	well defined	38.87	26.73
19	38	F	OL	Lt. Insular	2	wt	***	homo	6.37	well defined	46.64	38.08
20	39	F	OL	Lt. Frontal	2	mt	**	hetero	6.79	vague	62.99	56.89
21	40	M	OL	Rt. Frontal	2	mt	***	homo	6.02	well defined	23.11	19.11
22	44	F	OL	Rt. Frontal	2	mt	**	hetero	6.29	well defined	37.53	31.13
23	31	F	AA	Bil. Frontal	3	mt	***	hetero	6.45	vague	30.58	23.35
24	32	M	AA	Lt. Temporal—Occipital—Parietal—Frontal, Rt. Frontal	3	mt	***	hetero	6.40	vague	23.11	18.00
25	36	F	AA	Bil. Frontal	3	wt	***	hetero	6.55	vague	21.87	17.89
26	45	M	AA	Lt. Insular	3	mt	**	hetero	6.71	vague	46.42	34.89
27	45	M	AA	Lt. Parietal	3	wt	**	homo	5.12	vague	18.97	16.76
28	46	F	AA	Lt. Frontal—Temporal—Basal ganglia, Rt. Frontal	3	mt	***	hetero	5.39	vague	16.79	13.60
29	62	F	AA	Lt. Celebellum	3	wt	**	hetero	5.74	vague	19.81	16.97
30	62	F	AA	Lt. Temporal-Insular-Basal ganglia	3	wt	*	hetero	6.20	vague	29.22	20.56
31	68	M	AA	Rt. Frontal	3	mt	**	hetero	6.20	vague	24.12	17.00
32	70	M	AA	Rt. Frontal—Insular—Temporal	3	wt	**	hetero	5.72	vague	17.53	14.32
33	71	F	AA	Lt. Parietal	3	wt	***	hetero	5.70	vague	26.78	21.93
34	75	M	AA	Rt. Frontal	3	wt	***	hetero	5.41	well defined	26.93	18.44
35	78	M	AA	Corpus callosum	3	wt	***	homo	5.06	vague	15.36	12.65
36	79	M	AA	Lt. Frontal	3	wt	***	homo	5.04	vague	17.59	12.04
37	79	M	AA	Lt. Occipital	3	wt	***	hetero	5.67	well defined	23.60	19.65
38	38	M	AO	Rt. Frontal—Insular—Temporal	3	wt	*	hetero	5.88	vague	26.70	19.21
39	19	M	AOA	Lt. Frontal	3	mt	**	hetero	6.43	well defined	51.18	36.13
40	26	F	AOA	Bil. Frontal—Corpus callosum	3	mt	**	hetero	6.92	well defined	60.75	47.52
41	27	M	AOA	Bil. Frontal—Corpus callosum	3	wt	**	hetero	7.12	well defined	58.59	40.71
42	29	F	AOA	Lt. Insular	3	wt	**	hetero	6.23	well defined	42.86	34.06
43	32	F	AOA	Rt. Frontal	3	wt	***	hetero	6.25	well defined	31.09	24.00
44	35	M	AOA	Lt. Temporal	3	wt	**	hetero	6.25	well defined	34.18	28.75
45	39	F	AOA	Rt. Frontal	3	mt	**	hetero	6.95	well defined	64.04	50.46
46	39	F	AOA	Rt. Frontal—Insular—Temporal	3	mt	***	hetero	6.31	vague	23.88	18.00
47	41	M	AOA	Rt. Frontal	3	mt	**	hetero	7.21	well defined	52.28	35.51
48	52	M	AOA	Rt. Frontal	3	mt	**	hetero	7.14	well defined	42.09	29.43
49	76	M	AOA	Lt. Parietal	3	mt	**	hetero	6.34	vague	31.47	22.56
50	88	F	AOA	Lt. Parietal	3	mt	**	hetero	7.01	vague	46.96	39.12

**Abbreviations:** Age: M = male; F = female. Dignosis: DA = Diffuse astrocytoma; GG = Ganglioglioma; OA = Ogligoastrocytoma; OL = Oligodendroglioma; AA = Anaplastic astrocytoma; AO = Anaplastic oligodendroglioma, AOA = Anaplastic oligastrocytoma. Location: Rt = Right; Lt = Left. *IDH* status: wt = wild type; mt = mutant.

* = determined by immunohistochemistry,

** = determined by Sanger sequencing,

*** = determined by pyrosequencing

### *IDH1* mutation detection

Mutation hotspots at codon 132 of the *IDH1* gene were screened using DNA sequencing (pyrosequencing or Sanger sequencing) except for three cases that were determined using immunohistochemistry. For pyrosequencing, the following oligonucleotide primers were used for amplification; forward primer: CAAAAATATCCCCCGGCTTG, reverse primer: CAACATGACTTACTTGATCCCC (biotinylated at the 5`end). The primers for pyrosequencing were designed to correspond to the region immediately upstream of the hotspot for *IDH1*: ACCTATCATCATAGGT, and the pyrosequencing assays were designed to detect all known mutations at *IDH1* R132. Templates were prepared by PCR amplification from genomic DNA with the primer pairs described above, and pyrosequencing was carried out and analyzed using the AQ assay of a PyroMark Q96 (version 2.5.7) on a PyroMark ID pyrosequencer (Qiagen, Tokyo, Japan) as per the manufacturer’s recommendation [14]. For Sanger sequencing, *IDH1* exon 4 containing codon 132 was amplified from genomic DNAs by PCR with gene specific primers; forward primer: AATGAGCTCTATATGCCATCACTG, reverse primer: TTCATACCTTGCTTAATGGGTGT. The PCR products were purified using the QIAquick Gel Extraction Kit (Qiagen, Venlo, Natherlands), and were then sequenced using the sequencing primer GCCATCACTGCAGTTGTAGGTTA and the BigDye^®^Terminator V1.1 Cycle Sequencing Kit (Thermo Fisher Scientific, Waltham, MA) using the ABI 3130xL Genetic Analyzer (Thermo Fisher Scientific) [15]. For immunohistochemical analysis of *IDH1* mutation, the anti-human *IDH1* R132H antibody from dianova (Hamburg, Germany) was used on a formalin-fixed paraffin-embedded tissue (FFPET) according to the manufacturer’s recommended protocol [16].

### MRI and image format conversion

**Fig 1:**
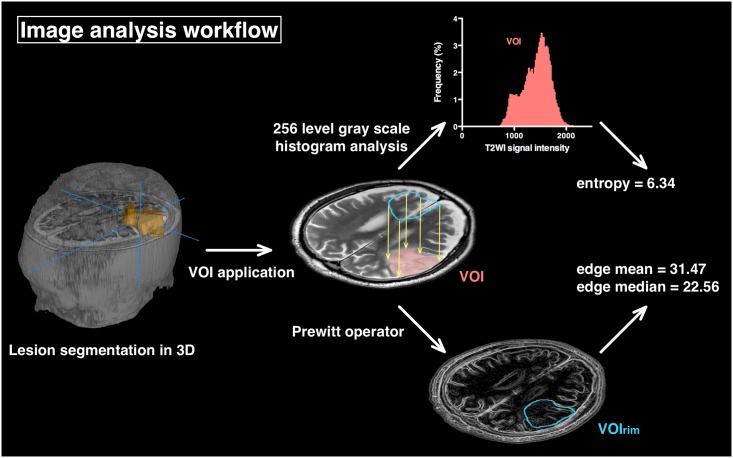
Image analysis workflow. The workflow for image analysis is presented. A high-intensity lesion on T2WI was first segmented in 3-dimensions, creating a voxels-of-interest (VOI). This VOI was applied to the original T2WI in a 256 level gray scale in order to calculate the Shannon entropy of the entire VOI. After the original T2WI was filtered using Prewitt filtering, the rim of the VOI (VOIrim) was applied to the edge enhanced image and the sharpness of the lesion border was calculated, reporting the edge mean and edge median values of the VOIrim.

All patients were studied using either a 1.5- or 3.0-T MRI scanner prior to surgery. T1-weighted imaging with and without gadolinium enhancement, T2-weighted (T2WI), and Fluid attenuation inversion recovery (FLAIR) images were acquired in all cases for delineation of tumors. T2WI was used for further analysis. Digital Imaging and COmmunication in Medicine (Dicom) format images were converted into Neuroimaging Informatics Technology Initiative (NIfTI) format using MRIConvert (http://lcni.uoregon.edu/downloads/mriconvert) and were subsequently analyzed by an in-house software developed on Matlab R2015 (MathWorks, Natick, MA). The developed software is capable of creating voxels-of-interest (VOI) in three dimensions from the original NIfTI data by manual segmentation. High intensity lesions on T2WI were manually segmented to create a 3D VOI for all of the cases (Fig 1). Diffuse high intensity lesions surrounding the presumed tumor core were all included in the VOI.

### Lesion heterogeneity analysis on T2WI by Shannon entropy

**Fig 2:**
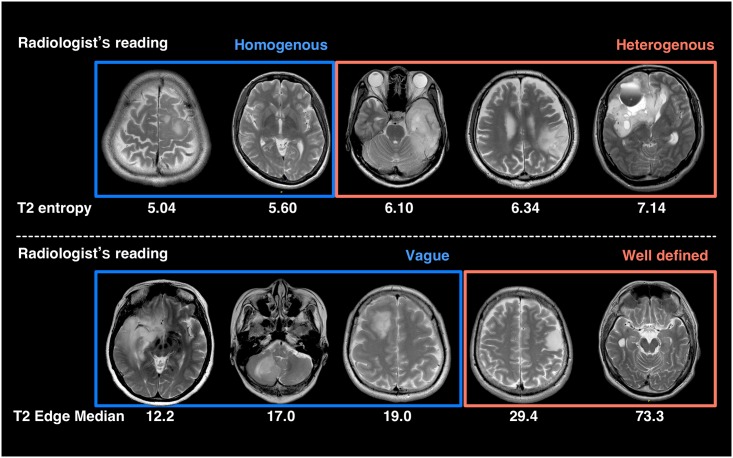
Representative cases. Representative cases that illustrates the relationship between radiologist’s readings and calculated texture metrics are shown. The upper panel shows the heterogeneity of the lesion assessed by T2 entropy and the lower shows tumor boarder sharpness assessed by Prewitt filtering.

Shannon entropy is a metric that is considered to reflect the amount of “information” contained within a message. This concept can be translated into the diversity of the image in a gray scale medical image such as MRI. A more diverse image, which means a more heterogeneous image will present higher Shannon entropy [17]. As gray scale medical images including MRI are presented in 256 (8 bit) levels, the lowest and highest intensity values of the whole image were determined and those MR signal intensities were converted into 256 levels. Next, a histogram of the predefined 256 levels within the VOI was analyzed, which enabled calculation of the Shannon entropy of the VOI. The Shannon entropy is defined by the following equation.

$$S=-\sum_{i=0}^{255}p_{i}{\text{log}}_{2}p_{i}$$

where *p*_*i*_ stands for the frequency of the gray scale level *i*. All of the images were separately read by a neuro-radiologist who was blinded to the calculated Shannon entropy and were classified as either homogenous or heterogeneous (Table 1). Representative cases are shown in Fig 2.

### Lesion border sharpness analysis on T2WI by Prewitt filtering

P rewitt filtering is a commonly used image filtering for detection of contours within an image [18]. It is a first order differentiation operator that approximates the gradient of the image intensity. The following matrices are applied to the original image to perform first order differentiation in both the horizontal (G*x*) and the vertical (G*y*) direction of the image,

$${\text{G}}_{x}=\left[\begin{array}{ccc} -1 & 0 & +1\\ -1 & 0 & +1\\ -1 & 0 & +1 \end{array}\right]*\text{A}$$

$${\text{G}}_{y}=\left[\begin{array}{ccc} -1 & -1 & -1\\ 0 & 0 & 0\\ +1 & +1 & +1 \end{array}\right]*\text{A}$$

where G*x* and G*y* stand for the horizontal and vertical gradient of the image, respectively, and A stands for the original two dimensional gray scale image. By use of both horizontal and vertical gradients of the image, the gradient magnitude can be calculated as follows,

$$\text{G}=\sqrt{{\text{G}}_{x}^{2}+{\text{G}}_{y}^{2}}$$

After T2WI is converted into a 256 level gray scale image with the lowest signal intensity set to 0 and the highest to 255, it can be converted into an edge contrasted image by applying the above mentioned image filtering technique (Fig 1). As this newly created image represents the magnitude of the gradient within the image that was originally a 256-level gray scale, the values that constitute the image can be directly compared between each case. The sharpness of the lesion border was then calculated by first extracting the rim of the VOI (VIO_rim_) that represents the lesion and then applying VIO_rim_ to the Prewitt filtered image, which allows calculation of the mean (Edge mean) and median (Edge median) magnitude of the gradient within VIO_rim_ (Fig 1 and Table 1). All of the images were separately read by a neuro-radiologist who was blinded to the calculated Edge mean and median, and were classified as either vague or well defined (Table 1). Representative cases are shown in Fig 2.

### Statistical analysis

Statistical analysis was performed using Prism 5 for Mac OS X (GraphPad Software, Inc. La Jolla, CA). Student’s *t*-test was used for 2-group comparisons. A receiver Operating characteristic (ROC) curve was created to test the sensitivity and specificity of the calculated values that match the subjective reading of the MRI by neuro-radiologists. A *p* value < 0.05 was considered statistically significant. The area under the curve (AUC) was calculated for each ROC curve analysis to evaluate the statistical performance of each metric to discriminate the specified observations.

## Results

### Lesion heterogeneity and Shannon entropy on T2WI

**Fig 3:**
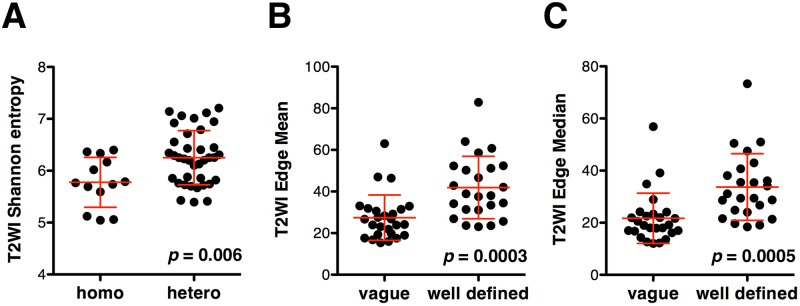
Comparison of image texture metrics and radiological readings. Lesions were defined by a neuro-radiologist who was blinded to the calculated values. Shannon entropy on T2WI was significantly higher for heterogeneous lesions than for homogenous lesions **(A)**. T2WI Edge mean **(B)** and median **(C)** values were both significantly higher for lesions with well-defined borders than for those with vague borders. Values are presented as mean ± 2SD.

**Fig 4:**
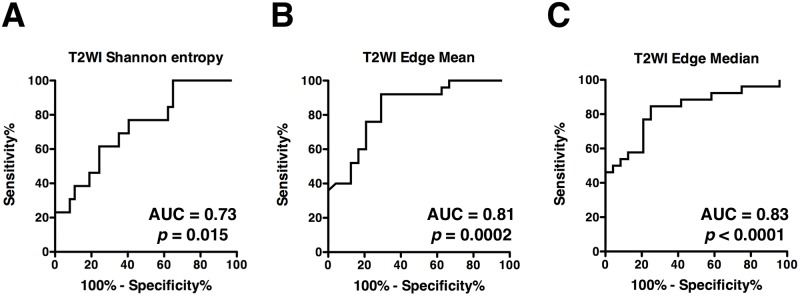
ROC analysis of image texture metrics. ROC curve analysis proved that Shannon entropy on T2WI was a reliable indicator for discrimination of homogenous and heterogeneous lesions identified by a neuro-radiologist **(A)**. ROC curve analysis also proved that both Edge mean **(B)** and median **(C)** values were promising indicators for discrimination of lesions with vague and well defined borders and both Edge mean and median values performed in a comparable manner.

First, Shannon entropy on T2WI was compared between homogenous and heterogeneous lesions as defined by a neuro-radiologist who was blinded to the calculated values. Heterogeneous lesions showed statistically higher Shannon entropy than homogenous lesions (Mean difference = 0.47, 95% confidence interval = 0.14 to 0.81, *p* = 0.006, Fig 3A). ROC curve analysis also proved that Shannon entropy on T2WI was a reliable indicator for discrimination of homogenous and heterogeneous lesions as identified by a neuro-radiologist (*p* = 0.015, AUC = 0.73, 95% confidence interval = 0.57 to 0.88, Fig 4A).

### Lesion border sharpness by Prewitt filtering on T2WI

Next, lesion border sharpness was calculated and Edge mean and Edge median values were obtained as described above. When these values were compared with the neuro-radiologist’s image readings, it was clearly shown that lesions with well-defined borders exhibited statistically higher Edge mean and Edge median values than those with vague lesion borders (Mean difference = 14.48, 95% confidence interval = 7.06 to 21.90, *p* = 0.0003 and Mean difference = 11.99, 95% confidence interval = 5.57 to 18.40, *p* = 0.0005 respectively, Fig 3B and 3C). ROC curve analysis also proved that both Edge mean and median values were promising indicators for discrimination of lesions with vague and well defined borders and both Edge mean and median values performed in a comparable manner (*p* = 0.0002, AUC = 0.81, 95% confidence interval = 0.69 to 0.93 and *p* < 0.0001, AUC = 0.83, 95% confidence interval = 0.71 to 0.94 respectively, Fig 4B and 4C).

### Lesion texture on T2WI and *IDH1* mutation status in WHO grade 2 and 3 gliomas

**Fig 5:**
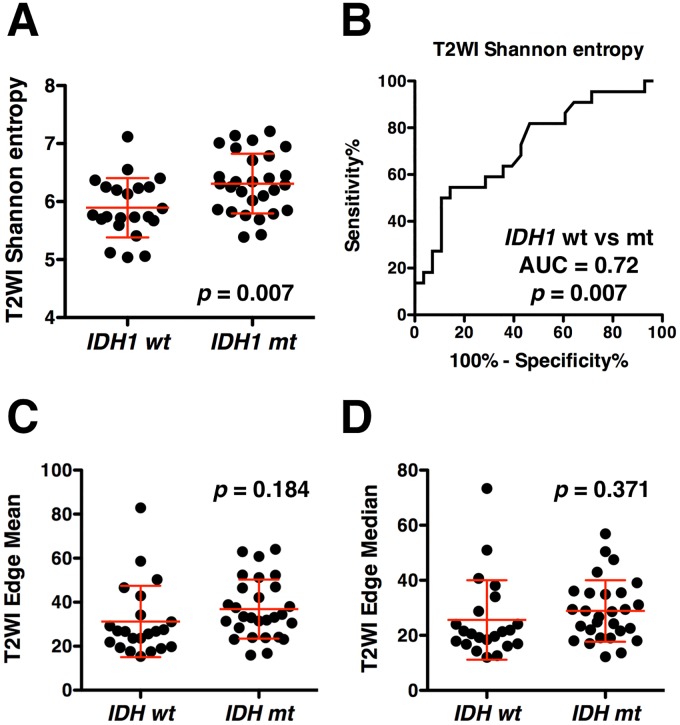
Lesion texture on T2WI and *IDH1* mutation status. *IDH1* wild type (wt) gliomas showed statistically lower Shannon entropy on T2WI than *IDH1* mutated (mt) gliomas **(A)**. This finding was confirmed by the fact that T2WI Shannon entropy showed an AUC of 0.72 in ROC curve analysis with a *p* value as low as 0.007 **(B)**. Lesion border sharpness evaluated by Prewitt filtering of the image could not predict the *IDH1* mutation status of the tumor **(C** and **D)**. Values are presented as mean ± 2SD for **(A) (C)** and **(D)**.

Finally, the above mentioned exploratory measurements were compared between *IDH1* wild type and mutated WHO grade 2 and 3 gliomas. *IDH1* wild type gliomas showed statistically lower Shannon entropy on T2WI than *IDH1* mutated gliomas (Mean difference = 0.42, 95% confidence interval = 0.12 to 0.71, *p* = 0.007, Fig 5A). This result can be interpreted as that *IDH1* wild type gliomas have a higher chance of exhibiting homogenous lesions than *IDH1* mutated gliomas. This finding was also confirmed by the fact that T2WI Shannon entropy showed an AUC of 0.72 in ROC curve (95% confidence interval = 0.58 to 0.87) analysis with a *p* value as low as 0.007 (Fig 5B). Lesion border sharpness, on the other hand, as evaluated by Prewitt filtering of the image, could not predict *IDH1* mutation status of the tumor. No difference was observed between *IDH1* wild type and mutated gliomas in the value of the Edge mean and median, which were both confirmed to reliably reflect the neuro-radiologist’s image reading (Fig 5C and 5D).

## Discussion

The relationship between the genetic status of a glioma and lesion presentation on radiological images such as MRI has been of great interest within the neuro-oncology community [5–7]. Pre-surgical identification of such biological characteristics of the tumor can contribute to surgical planning and determine whether the tumor should be biopsied or extensively removed. Several techniques have been reported to address this task, including the use of magnetic resonance spectroscopy (MRS) to detect 2-hydroxyglutarate (2-HG) as a metabolic product of *IDH1/2* mutation [5–7] or the use of ^11^C-methionine positron emission tomography for detection of 1p/19q co-deletion in WHO grade 2 and 3 gliomas [19]. Furthermore, MRI characteristics such as sharpness of the tumor border and heterogeneity of the lesion were reported to provide an image surrogate for identification of 1p/19q co-deletion status [13]. As large scale radiological image collection is now starting to be available during various clinical studies, development of high throughput image analyzing techniques is mandatory. Such high throughput image analysis has been mainly focused on voxel-based lesion location analysis, which is based on lesion mapping on a standard MR brain atlas. This type of analysis successfully elucidated the preferred locations of *IDH1/2* mutated and *MGMT* promoter methylated glioblastoma [8–10]. On the other hand, there have been few attempts at objective and high throughput analysis of lesion textures on MRI. As this radiological information might be beneficial for identifying genetic characteristics of the tumor, this report attempted to develop and validate two potential measureable indicators that are thought to reflect lesion heterogeneity and lesion border sharpness.

First, Shannon entropy was evaluated on T2WI, as we hypothesized that this metric would correlate with the magnitude of lesion heterogeneity. T2WI was chosen as the image set to be tested because T2WI has a dynamic range of image with the lowest signal intensity being 0 and the highest that is determined by cerebrospinal fluid. Since the highest and lowest signal intensity can always be determined by these two elements, calculation of Shannon entropy may be more reliable than calculation of other MR sequences such as T1WI or FLAIR, for both of which the highest signal intensity may not derive from constant anatomical structures. The results of the current study clearly showed that Shannon entropy on T2WI correlated well with the neuro-radiologist’s readings of the image (Figs 3A and 4A). This result suggested that Shannon entropy on T2WI can be further utilized as a reliable indicator for objective and high throughput image analysis.

Next, Prewitt filtering was evaluated for detection of tumor border sharpness. A Prewitt filter (operator) is a first order differentiation operator that approximates the gradient of the image intensity. Taking this theoretical background into consideration, values obtained from Prewitt filtered T2WI should correlate with lesion border sharpness, i.e., sharp border lesions should have higher values and vice versa. Again, the current study was able to prove that this type of image analysis correlated well with the neuro-radiologist’s readings of the image (Figs 3B, 3C, 4B and 4C).

Finally, correlations between the above two measurements of T2WI Shannon entropy and the median value within the lesion rim of Prewitt filtered images, and the *IDH1* mutation status of the lesion, was tested as an exploratory attempt to use these novel image analysis metrics for an objective and high throughput image analyzing study. The results shown in Fig 5A indicated that lesion heterogeneity is different between *IDH1* mutated and wild type gliomas. On the other hand, lesion border sharpness as assessed by Prewitt filtered images was not affected by this type of genetic mutation of the tumor. These results suggest that, while Shannon entropy on T2WI can be used as an image surrogate for *IDH1* mutation status of the tumor (Fig 5B), tumor border sharpness is not a promising image metric for this purpose (Fig 5C and 5D).

In conclusion, the current study introduced two image metrics that reflect lesion texture described on T2WI of gliomas. These two metrics were validated by readings of a neuro-radiologist who was blinded to the results. Although the described method requires manual segmentation of the tumor which contradicts analyzing large number of images, it should be noted that images can be evaluated in an objective and numerical manner compared to mere radiological reading of the images. Furthermore, once the region of interest (ROI) is defined, that ROI can be used to analyze other images such as T1-weighted or Fluid-attenuated inversion recovery (FLAIR) images registered on to T2WI, which approach is in line with a “radionomic” analysis of large scale samples.
